# Data on the expression and insulin-stimulated phosphorylation of IRS-1 by miR-96 in L6-GLUT4myc myocytes

**DOI:** 10.1016/j.dib.2017.10.054

**Published:** 2017-10-28

**Authors:** Won-Mo Yang, Kyung-Ho Min, Yi-Seul Son, Se-Whan Park, Wan Lee

**Affiliations:** a Department of Biochemistry, Dongguk University College of Medicine, Gyeongju-si, Gyeongsangbuk-do 38067, Republic of Korea; b Endocrine Channelopathy, Channelopathy Research Center, Dongguk University College of Medicine, Goyang-si, Gyeonggi-do 10326, Republic of Korea

**Keywords:** MicroRNAs, miR-96, Myocyte, IRS-1, INSR

## Abstract

Diets containing a high saturated fatty acid (SFA) increase the risk of metabolic diseases, and microRNAs (miRNAs) induced by SFA have been implicated in the pathogenesis of insulin resistance and type 2 diabetes. In a previous report, miR-96 is found to be upregulated by SFA and involved in the suppression of insulin signaling intermediates, leading to insulin resistance in hepatocytes (Yang et al., 2016) [1]. This article presents the accompanying data collected from L6-GLUT4myc myocytes to determine the effects of miR-96 on insulin signaling in skeletal muscle cells. The transfection of miR-96 decreased the expression of IRS-1 in myocytes. Accordingly, miR-96 inhibited the insulin-stimulated phosphorylation of IRS-1, which led to an impairment of insulin signaling. More detailed analysis and understanding of the roles of miR-96 in diet-induced insulin resistance can be found in "Induction of miR-96 by dietary saturated fatty acids exacerbates hepatic insulin resistance through the suppression of INSR and IRS-1" (Yang et al., 2016) [1].

**Specifications Table**

**Table:** 

Subject area	*Cell Biology, Biochemistry*
More specific subject area	*MicroRNA, Metabolism, Obesity*
Type of data	*Figures and text*
How data was acquired	*Analysis of RT-PCR, qRT-PCR, and immunoblotting*
Data format	*Analyzed*
Experimental factors	*Transfection of miR-96, Treatment of insulin, Analysis of the expression and phosphorylation of insulin signaling intermediates*
Experimental features	*L6-GLUT4myc myocytes were transfected with scRNA or miR-96 mimic. For insulin stimulation, 100 nM of insulin was treated during the last 30 min of incubation.*
Data source location	*Dongguk University School of Medicine Gyeongju-si, Gyeongsangbuk-do 38067, Korea*
Data accessibility	*The data are available with this article*

**Value of the data**

• The data revealed the IRS-1 suppression by miR-96 in L6-GLUT4myc myocytes.
• The data are useful for understanding the regulatory mechanism of IRS-1 expression by miR-96.
• The data can be compared with the molecular function of miR-96 between myocytes or other tissue types.
• The modulation of miR-96 can be applied to design diagnostic and therapeutic strategies for metabolic diseases.

## 1 Data

**Fig. 1:**
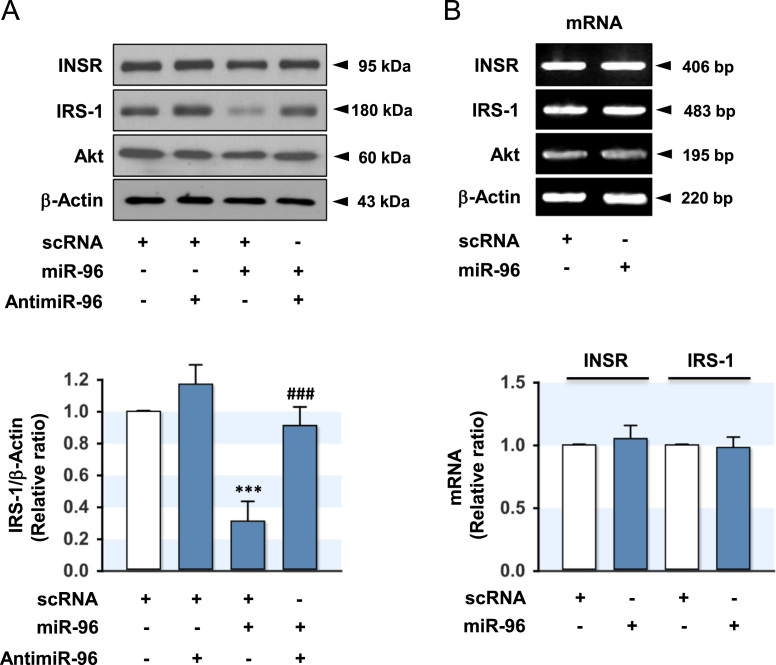
Effect of miR-96 on the INSR and IRS-1 expression. (A) L6-GLUT4myc myocytes were transfected with scRNA (100 nM), AntimiR-96 (100 nM), and/or miR-96 (100 nM) mimic. After 48 h transfection, expression of the insulin signaling intermediates was analyzed by immunoblotting. Representative immunoblots from five independent experiments and analyzed densitometry were shown in A. The immunoblot intensity of IRS-1 was normalized to the amount of β-Actin. (B) L6-GLUT4myc myocytes were transfected with 200 nM of miR-96 mimic or scRNA control. The mRNA levels were analyzed at 24 h after transfection by RT-PCR (upper panel) and *q*RT-PCR (lower panel). Values are the means ± SEM. ***, P < 0.001 vs. scRNA control; ###, p < 0.001 scRNA + miR-96 vs. AntimiR-96 + miR-96.

**Fig. 2:**
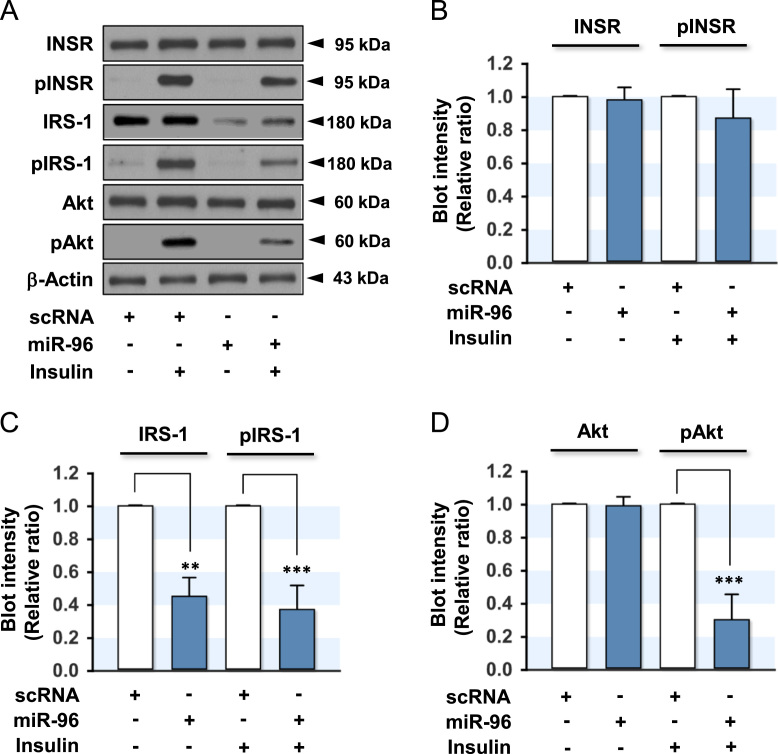
Effect of miR-96 on the expression and phosphorylation of insulin signaling molecules. L6-GLUT4myc myocytes were transfected with the scRNA (200 nM) or miR-96 (200 nM) mimic. After 48 h transfection, the cells were incubated in the presence or absence of insulin (100 nM) for 30 min and subjected to immunoblotting. (A) Representative immunoblots obtained from L6-GLUT4myc myocytes are shown in A. (B) The expression and phosphorylation of INSR (pINSR) were normalized to the amount of INSR. (C) The expression and phosphorylation of IRS-1 (pIRS-1) were normalized to the amount of β-Actin. (D) The protein expression of Akt was normalized to the amount of β-Actin. The level of Akt phosphorylation (pAkt2) was normalized to the amount of Akt. The values are the relative ratio, where the intensity of the scRNA control was set to one, and expressed as the means ± SEM from three independent experiments. *, P < 0.05; **, P < 0.01; ***, P < 0.001 vs. scRNA control.

Certain miRNAs targeting the molecules in the insulin signal transduction pathway are dysregulated in saturated fatty acid (SFA)-induced obesity, and these miRNAs contribute to the development of insulin resistance in the liver and skeletal muscle [2], [3]. The upregulation of miR-96 in SFA palmitate-treated hepatocytes was recently reported to downregulate IRS-1 expression by binding to its 3′UTR regions on mRNA, leading to hepatic insulin resistance [1]. This article presents accompanying data to examine further the effect of miR-96 in the skeletal muscle. The scRNA control or miR-96 mimic was transfected into the L6-GLUT4myc myocytes, as described in the Section 2, and the expression of insulin signaling intermediates, such as INSR, IRS-1 and Akt2, were analyzed. As shown in Fig. 1A, transfection of the miR-96 mimic decreased the protein level of IRS-1 compared to the scRNA control, whereas the protein levels of INSR and Akt were unaffected. Co-transfection with AntimiR-96 abolished the suppressive effect of miR-96 on IRS-1 expression. In addition, the mRNA levels of INSR, IRS-1, and Akt remained unaffected by miR-96 in myocytes (Fig. 1B). Therefore, this data indicates that miR-96 suppresses the expression of IRS-1 at the post-transcriptional level in L6-GLUT4myc myocytes. Furthermore, the insulin-stimulated phosphorylation of insulin signaling molecules, such as INSR, IRS-1 and Akt, was determined in miR-96-transfected myocytes (Fig. 2). The transfection of miR-96 mimic in myocytes suppressed the insulin-stimulated phosphorylation of IRS-1 and its down-stream target, Akt, significantly (Fig. 2A, C, D). Based on the expression level, the inhibitory effect of miR-96 on the insulin-stimulated phosphorylation of IRS-1 was attributed mainly to the lower level in IRS-1 expression. On the other hand, the expression and phosphorylation of INSR in myocytes were unaffected by the transfection of the miR-96 mimic (Fig. 2A, B) because the 3′UTR of INSR in L6-GLUT4myc myocytes, which was derived originally from the rat skeletal muscle, does not have any binding site for miR-96. Therefore, the induction of miR-96 leads to an impairment of insulin signaling in myocytes through the repression of IRS-1 expression. Further analysis of the data and discussion of the implication of miR-96 in insulin resistance and the pathogenesis of type 2 diabetes are presented in "Induction of miR-96 by dietary saturated fatty acids exacerbates hepatic insulin resistance through the suppression of INSR and IRS-1" [1].

## 2 Experimental design, materials and methods

### 2.1 Cells culture and insulin treatment

L6-GLUT4myc myocytes derived from rat skeletal muscle were purchased from Kerafast, Inc. (Boston, MA, USA). The cells were cultured in MEMα with 10% FBS and 1% penicillin-streptomycin (Gibco) in an atmosphere containing 5% CO_2_ at 37 °C, as described elsewhere [4]. The undifferentiated myocytes from passages 4 to 10 were used for the following experiments. For insulin stimulation, the cells were cultured in serum-free medium for the last 5 h of the experiment, which was followed by a treatment with insulin (100 nM) for the last 30 min.

### 2.2 Transfection of miRNA mimics

L6-GLUT4myc myocytes were transfected with the 100 or 200 nM mimics of miR-96, AntimiR-96 and/or scrambled control miRNA (scRNA) using G-fectin (Genolution, Seoul, Korea) according to the manufacturer's instructions. The miRNA mimics and scRNA were purchased from Genolution.

### 2.3 RNA extraction, RT-PCR, and qRT-PCR

The total RNA from the L6-GLUT4myc myocytes was extracted using a miRNeasy Mini Kit (Qiagen). To analyze mRNA expression, cDNA was synthesized from the total cellular RNA (1 μg) using the miScript II RT Kit (Qiagen). The levels of mRNA (INSR, IRS-1, Akt, and β-Actin) were measured by RT-PCR or *q*RT-PCR using a GoTaq Green Master Mix (Promega) or miScript SYBR Green PCR Kit (Qiagen), respectively. The primers (Bionics, Seoul, Korea) and PCR conditions are described elsewhere [4]. The intensity data of *q*RT-PCR were analyzed using the advanced relative quantification method in Light-Cycler 480 software (Roche Diagnostics). β-Actin was applied as an internal control for the expression of the mRNAs.

### 2.4 Cell lysis, immunoblotting and antibodies

L6-GLUT4myc myocytes were washed three times with ice-cold PBS and lysed using a lysis buffer (ice-cold PBS containing 1% Triton X-100, phosphatase inhibitor cocktail II, and 0.2 mM PMSF) by homogenization. The lysates were mixed with 2X Laemmli buffer, and heated for 10 min at 100 °C. Gel electrophoresis was carried out by SDS–PAGE on 10 or 8% resolving gels, transferred and immunoblotted with various antibodies. The anti-IRS-1 antibody was obtained from Upstate Biotechnology (Lake Placid, NY, US). The antibodies against phospho-tyrosine-IRS-1 (Tyr632) and β-actin were purchased from Santa Cruz Biotechnology (Santa Cruz, CA, US). The antibodies against INSR, phospho-tyrosine-INSR (Tyr1361), Akt, phospho-serine-Akt (Ser473) were obtained from Cell Signaling Technology (Danvers, MA, US). ECL Western Blotting Detection Reagents from GE Healthcare (Buckinghamshire, UK) were used to visualize the immunoblot. The intensities of the immunoblots were determined by densitometry using an Alpha Imager HP scanning system (Alpha Innotech, San Leandro, CA, US).

### 2.5 Statistical analysis

The data are expressed as a mean ± SEM. from at least three independent sets of experiments. The significance of the difference was tested using a Student's *t*-test for unpaired data.
